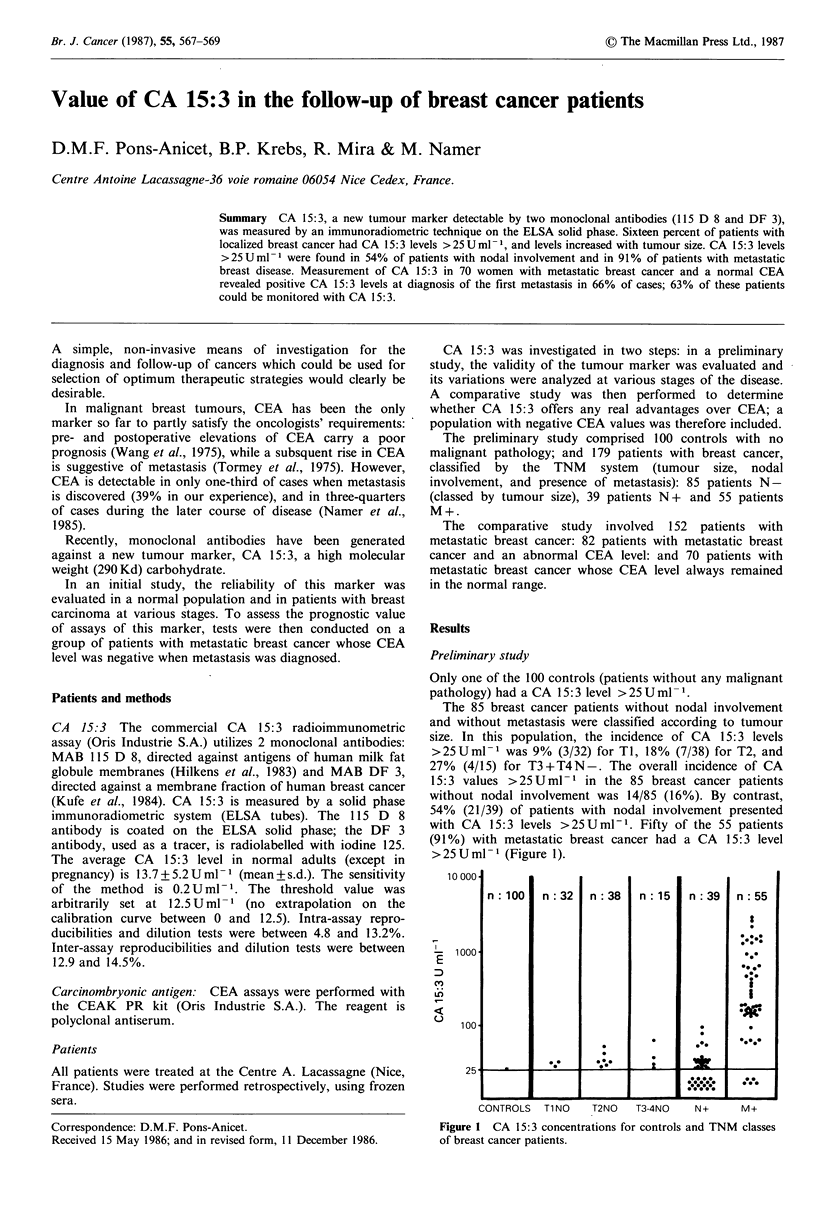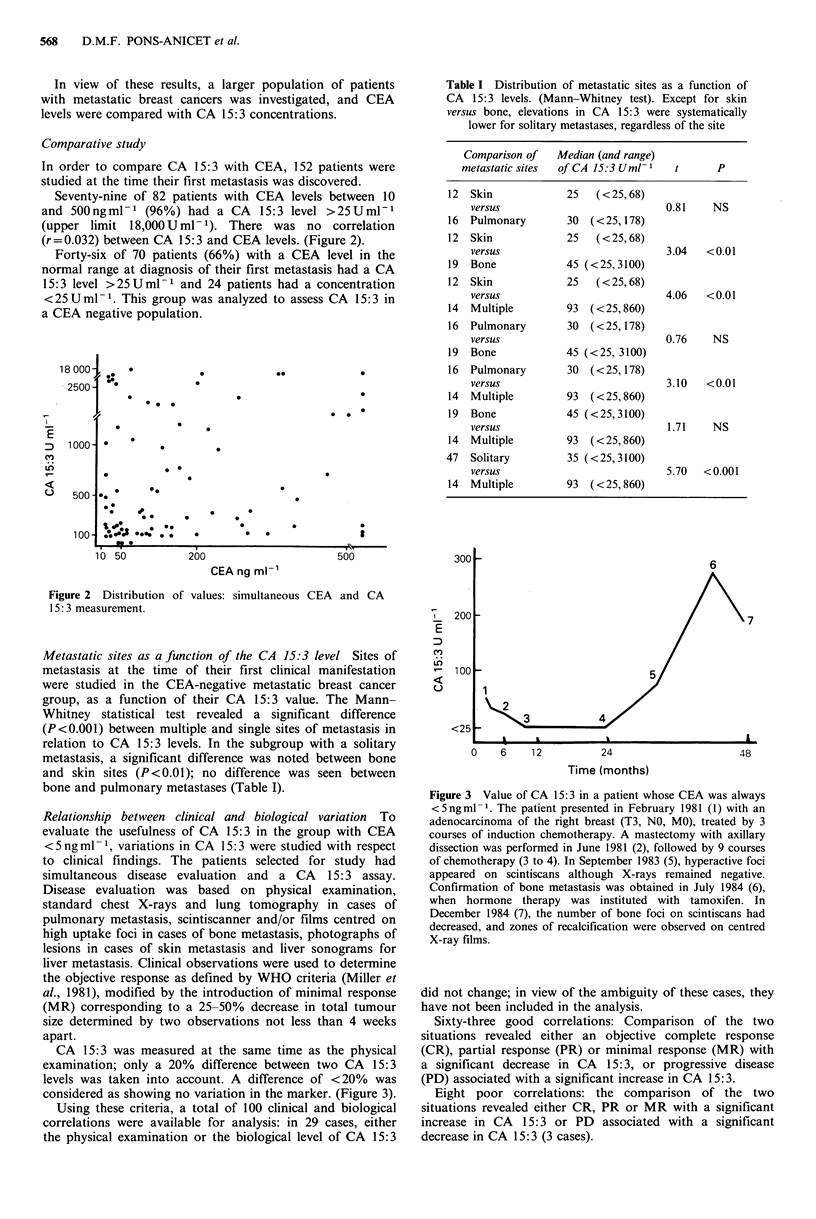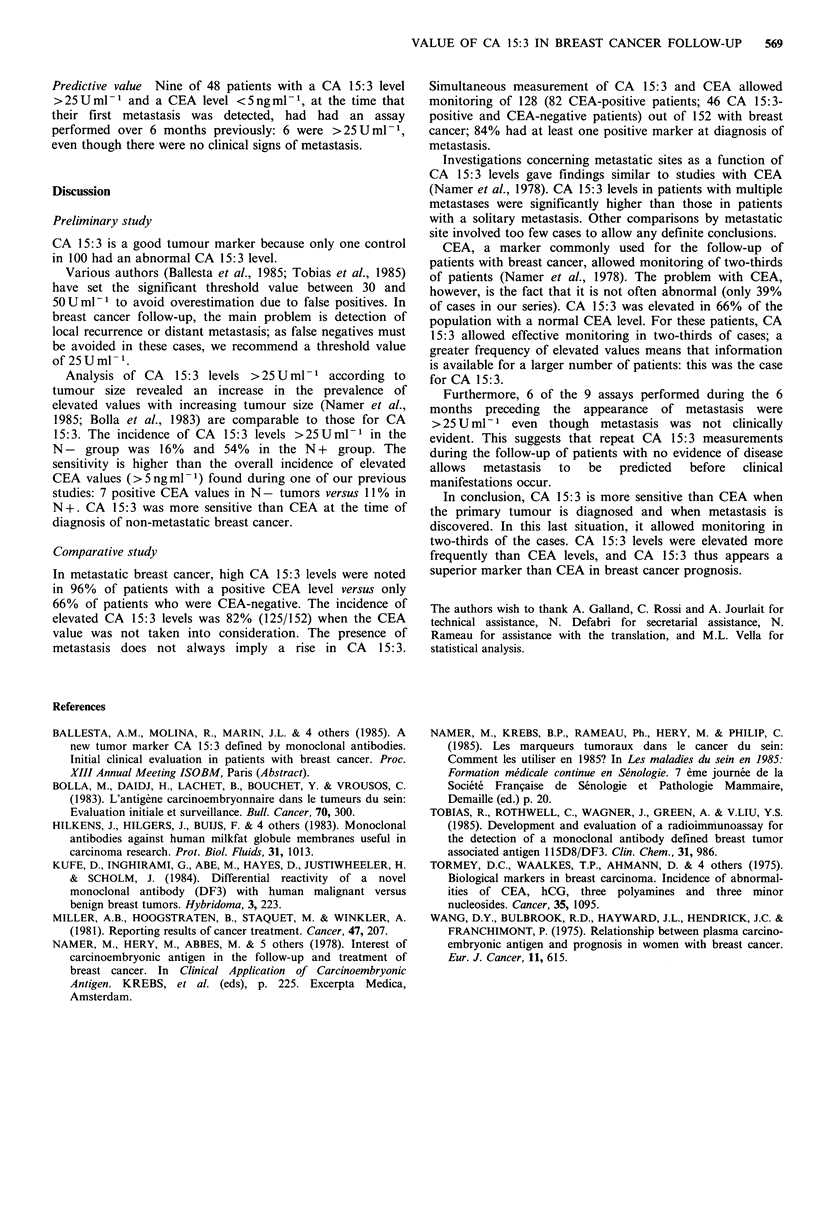# Value of CA 15:3 in the follow-up of breast cancer patients.

**DOI:** 10.1038/bjc.1987.115

**Published:** 1987-05

**Authors:** D. M. Pons-Anicet, B. P. Krebs, R. Mira, M. Namer

## Abstract

CA 15:3, a new tumour marker detectable by two monoclonal antibodies (115 D 8 and DF 3), was measured by an immunoradiometric technique on the ELSA solid phase. Sixteen percent of patients with localized breast cancer had CA 15:3 levels greater than 25 U ml-1, and levels increased with tumour size. CA 15:3 levels greater than 25 U ml-1 were found in 54% of patients with nodal involvement and in 91% of patients with metastatic breast disease. Measurement of CA 15:3 in 70 women with metastatic breast cancer and a normal CEA revealed positive CA 15:3 levels at diagnosis of the first metastasis in 66% of cases; 63% of these patients could be monitored with CA 15:3.


 © The Macmillan Press Ltd., 1987

### Value of CA 15:3 in the follow-up of breast cancer patients

D.M.F. Pons-Anicet, B.P. Krebs, R. Mira & M. Namer

*Centre Antoine Lacassagne-36 voie romaine 06054 Nice Cedex, France.*

**Summary** CA 15:3, a new tumour marker detectable by two monoclonal antibodies (115 D 8 and DF 3), was measured by an immunoradiometric technique on the ELSA solid phase. Sixteen percent of patients with localized breast cancer had CA 15:3 levels $>25\,U\,ml^{-1}$, and levels increased with tumour size. CA 15:3 levels $>25\,U\,ml^{-1}$ were found in 54% of patients with nodal involvement and in 91% of patients with metastatic breast disease. Measurement of CA 15:3 in 70 women with metastatic breast cancer and a normal CEA revealed positive CA 15:3 levels at diagnosis of the first metastasis in 66% of cases; 63% of these patients could be monitored with CA 15:3.

A simple, non-invasive means of investigation for the diagnosis and follow-up of cancers which could be used for selection of optimum therapeutic strategies would clearly be desirable.

In malignant breast tumours, CEA has been the only marker so far to partly satisfy the oncologists' requirements: pre- and postoperative elevations of CEA carry a poor prognosis (Wang *et al.*, 1975), while a subsquent rise in CEA is suggestive of metastasis (Tormey *et al.*, 1975). However, CEA is detectable in only one-third of cases when metastasis is discovered (39% in our experience), and in three-quarters of cases during the later course of disease (Namer *et al.*, 1985).

Recently, monoclonal antibodies have been generated against a new tumour marker, CA 15:3, a high molecular weight (290 Kd) carbohydrate.

In an initial study, the reliability of this marker was evaluated in a normal population and in patients with breast carcinoma at various stages. To assess the prognostic value of assays of this marker, tests were then conducted on a group of patients with metastatic breast cancer whose CEA level was negative when metastasis was diagnosed.

### Patients and methods

*CA 15:3* The commercial CA 15:3 radioimmunometric assay (Oris Industrie S.A.) utilizes 2 monoclonal antibodies: MAB 115 D 8, directed against antigens of human milk fat globule membranes (Hilkens *et al.*, 1983) and MAB DF 3, directed against a membrane fraction of human breast cancer (Kufe *et al.*, 1984). CA 15:3 is measured by a solid phase immunoradiometric system (ELSA tubes). The 115 D 8 antibody is coated on the ELSA solid phase; the DF 3 antibody, used as a tracer, is radiolabelled with iodine 125. The average CA 15:3 level in normal adults (except in pregnancy) is $13.7\pm5.2\,U\,ml^{-1}$ (mean $\pm$ s.d.). The sensitivity of the method is $0.2\,U\,ml^{-1}$. The threshold value was arbitrarily set at $12.5\,U\,ml^{-1}$ (no extrapolation on the calibration curve between 0 and 12.5). Intra-assay reproducibilities and dilution tests were between 4.8 and 13.2%. Inter-assay reproducibilities and dilution tests were between 12.9 and 14.5%.

*Carcinombryonic antigen:* CEA assays were performed with the CEAK PR kit (Oris Industrie S.A.). The reagent is polyclonal antiserum.

### *Patients*

All patients were treated at the Centre A. Lacassagne (Nice, France). Studies were performed retrospectively, using frozen sera.

Correspondence: D.M.F. Pons-Anicet.
Received 15 May 1986; and in revised form, 11 December 1986.

CA 15:3 was investigated in two steps: in a preliminary study, the validity of the tumour marker was evaluated and its variations were analyzed at various stages of the disease. A comparative study was then performed to determine whether CA 15:3 offers any real advantages over CEA; a population with negative CEA values was therefore included.

The preliminary study comprised 100 controls with no malignant pathology; and 179 patients with breast cancer, classified by the TNM system (tumour size, nodal involvement, and presence of metastasis): 85 patients N− (classed by tumour size), 39 patients N+ and 55 patients M+.

The comparative study involved 152 patients with metastatic breast cancer: 82 patients with metastatic breast cancer and an abnormal CEA level: and 70 patients with metastatic breast cancer whose CEA level always remained in the normal range.

### Results

### *Preliminary study*

Only one of the 100 controls (patients without any malignant pathology) had a CA 15:3 level $>25\,U\,ml^{-1}$.

The 85 breast cancer patients without nodal involvement and without metastasis were classified according to tumour size. In this population, the incidence of CA 15:3 levels $>25\,U\,ml^{-1}$ was 9% (3/32) for T1, 18% (7/38) for T2, and 27% (4/15) for T3+T4N−. The overall incidence of CA 15:3 values $>25\,U\,ml^{-1}$ in the 85 breast cancer patients without nodal involvement was 14/85 (16%). By contrast, 54% (21/39) of patients with nodal involvement presented with CA 15:3 levels $>25\,U\,ml^{-1}$. Fifty of the 55 patients (91%) with metastatic breast cancer had a CA 15:3 level $>25\,U\,ml^{-1}$ (Figure 1).

**Figure 1** CA 15:3 concentrations for controls and TNM classes of breast cancer patients.

In view of these results, a larger population of patients with metastatic breast cancers was investigated, and CEA levels were compared with CA 15:3 concentrations.

*Comparative study*

In order to compare CA 15:3 with CEA, 152 patients were studied at the time their first metastasis was discovered.

Seventy-nine of 82 patients with CEA levels between 10 and 500 ng ml$^{-1}$ (96%) had a CA 15:3 level >25 U ml$^{-1}$ (upper limit 18,000 U ml$^{-1}$). There was no correlation ($r$=0.032) between CA 15:3 and CEA levels. (Figure 2).

Forty-six of 70 patients (66%) with a CEA level in the normal range at diagnosis of their first metastasis had a CA 15:3 level >25 U ml$^{-1}$ and 24 patients had a concentration <25 U ml$^{-1}$. This group was analyzed to assess CA 15:3 in a CEA negative population.

**Figure 2** Distribution of values: simultaneous CEA and CA 15:3 measurement.

*Metastatic sites as a function of the CA 15:3 level* Sites of metastasis at the time of their first clinical manifestation were studied in the CEA-negative metastatic breast cancer group, as a function of their CA 15:3 value. The Mann–Whitney statistical test revealed a significant difference ($P$<0.001) between multiple and single sites of metastasis in relation to CA 15:3 levels. In the subgroup with a solitary metastasis, a significant difference was noted between bone and skin sites ($P$<0.01); no difference was seen between bone and pulmonary metastases (Table I).

*Relationship between clinical and biological variation* To evaluate the usefulness of CA 15:3 in the group with CEA <5 ng ml$^{-1}$, variations in CA 15:3 were studied with respect to clinical findings. The patients selected for study had simultaneous disease evaluation and a CA 15:3 assay. Disease evaluation was based on physical examination, standard chest X-rays and lung tomography in cases of pulmonary metastasis, scintiscanner and/or films centred on high uptake foci in cases of bone metastasis, photographs of lesions in cases of skin metastasis and liver sonograms for liver metastasis. Clinical observations were used to determine the objective response as defined by WHO criteria (Miller *et al.*, 1981), modified by the introduction of minimal response (MR) corresponding to a 25–50% decrease in total tumour size determined by two observations not less than 4 weeks apart.

CA 15:3 was measured at the same time as the physical examination; only a 20% difference between two CA 15:3 levels was taken into account. A difference of <20% was considered as showing no variation in the marker. (Figure 3).

Using these criteria, a total of 100 clinical and biological correlations were available for analysis: in 29 cases, either the physical examination or the biological level of CA 15:3 did not change; in view of the ambiguity of these cases, they have not been included in the analysis.

**Table I** Distribution of metastatic sites as a function of CA 15:3 levels. (Mann-Whitney test). Except for skin *versus* bone, elevations in CA 15:3 were systematically lower for solitary metastases, regardless of the site

| Comparison of metastatic sites | | Median (and range) of CA 15:3 U ml$^{-1}$ | $t$ | $P$ |
|---|---|---|---|---|
| 12 | Skin | 25 (<25, 68) | | |
| | *versus* | | 0.81 | NS |
| 16 | Pulmonary | 30 (<25, 178) | | |
| 12 | Skin | 25 (<25, 68) | | |
| | *versus* | | 3.04 | <0.01 |
| 19 | Bone | 45 (<25, 3100) | | |
| 12 | Skin | 25 (<25, 68) | | |
| | *versus* | | 4.06 | <0.01 |
| 14 | Multiple | 93 (<25, 860) | | |
| 16 | Pulmonary | 30 (<25, 178) | | |
| | *versus* | | 0.76 | NS |
| 19 | Bone | 45 (<25, 3100) | | |
| 16 | Pulmonary | 30 (<25, 178) | | |
| | *versus* | | 3.10 | <0.01 |
| 14 | Multiple | 93 (<25, 860) | | |
| 19 | Bone | 45 (<25, 3100) | | |
| | *versus* | | 1.71 | NS |
| 14 | Multiple | 93 (<25, 860) | | |
| 47 | Solitary | 35 (<25, 3100) | | |
| | *versus* | | 5.70 | <0.001 |
| 14 | Multiple | 93 (<25, 860) | | |

**Figure 3** Value of CA 15:3 in a patient whose CEA was always <5 ng ml$^{-1}$. The patient presented in February 1981 (1) with an adenocarcinoma of the right breast (T3, N0, M0), treated by 3 courses of induction chemotherapy. A mastectomy with axillary dissection was performed in June 1981 (2), followed by 9 courses of chemotherapy (3 to 4). In September 1983 (5), hyperactive foci appeared on scintiscans although X-rays remained negative. Confirmation of bone metastasis was obtained in July 1984 (6), when hormone therapy was instituted with tamoxifen. In December 1984 (7), the number of bone foci on scintiscans had decreased, and zones of recalcification were observed on centred X-ray films.

Sixty-three good correlations: Comparison of the two situations revealed either an objective complete response (CR), partial response (PR) or minimal response (MR) with a significant decrease in CA 15:3, or progressive disease (PD) associated with a significant increase in CA 15:3.

Eight poor correlations: the comparison of the two situations revealed either CR, PR or MR with a significant increase in CA 15:3 or PD associated with a significant decrease in CA 15:3 (3 cases).

*Predictive value* Nine of 48 patients with a CA 15:3 level $>25\,U\,ml^{-1}$ and a CEA level $<5\,ng\,ml^{-1}$, at the time that their first metastasis was detected, had had an assay performed over 6 months previously: 6 were $>25\,U\,ml^{-1}$, even though there were no clinical signs of metastasis.

### Discussion

### *Preliminary study*

CA 15:3 is a good tumour marker because only one control in 100 had an abnormal CA 15:3 level.

Various authors (Ballesta *et al.*, 1985; Tobias *et al.*, 1985) have set the significant threshold value between 30 and $50\,U\,ml^{-1}$ to avoid overestimation due to false positives. In breast cancer follow-up, the main problem is detection of local recurrence or distant metastasis; as false negatives must be avoided in these cases, we recommend a threshold value of $25\,U\,ml^{-1}$.

Analysis of CA 15:3 levels $>25\,U\,ml^{-1}$ according to tumour size revealed an increase in the prevalence of elevated values with increasing tumour size (Namer *et al.*, 1985; Bolla *et al.*, 1983) are comparable to those for CA 15:3. The incidence of CA 15:3 levels $>25\,U\,ml^{-1}$ in the N− group was 16% and 54% in the N+ group. The sensitivity is higher than the overall incidence of elevated CEA values ($>5\,ng\,ml^{-1}$) found during one of our previous studies: 7 positive CEA values in N− tumors *versus* 11% in N+. CA 15:3 was more sensitive than CEA at the time of diagnosis of non-metastatic breast cancer.

### *Comparative study*

In metastatic breast cancer, high CA 15:3 levels were noted in 96% of patients with a positive CEA level *versus* only 66% of patients who were CEA-negative. The incidence of elevated CA 15:3 levels was 82% (125/152) when the CEA value was not taken into consideration. The presence of metastasis does not always imply a rise in CA 15:3. Simultaneous measurement of CA 15:3 and CEA allowed monitoring of 128 (82 CEA-positive patients; 46 CA 15:3-positive and CEA-negative patients) out of 152 with breast cancer; 84% had at least one positive marker at diagnosis of metastasis.

Investigations concerning metastatic sites as a function of CA 15:3 levels gave findings similar to studies with CEA (Namer *et al.*, 1978). CA 15:3 levels in patients with multiple metastases were significantly higher than those in patients with a solitary metastasis. Other comparisons by metastatic site involved too few cases to allow any definite conclusions.

CEA, a marker commonly used for the follow-up of patients with breast cancer, allowed monitoring of two-thirds of patients (Namer *et al.*, 1978). The problem with CEA, however, is the fact that it is not often abnormal (only 39% of cases in our series). CA 15:3 was elevated in 66% of the population with a normal CEA level. For these patients, CA 15:3 allowed effective monitoring in two-thirds of cases; a greater frequency of elevated values means that information is available for a larger number of patients: this was the case for CA 15:3.

Furthermore, 6 of the 9 assays performed during the 6 months preceding the appearance of metastasis were $>25\,U\,ml^{-1}$ even though metastasis was not clinically evident. This suggests that repeat CA 15:3 measurements during the follow-up of patients with no evidence of disease allows metastasis to be predicted before clinical manifestations occur.

In conclusion, CA 15:3 is more sensitive than CEA when the primary tumour is diagnosed and when metastasis is discovered. In this last situation, it allowed monitoring in two-thirds of the cases. CA 15:3 levels were elevated more frequently than CEA levels, and CA 15:3 thus appears a superior marker than CEA in breast cancer prognosis.

The authors wish to thank A. Galland, C. Rossi and A. Jourlait for technical assistance, N. Defabri for secretarial assistance, N. Rameau for assistance with the translation, and M.L. Vella for statistical analysis.

### References

BALLESTA, A.M., MOLINA, R., MARIN, J.L. & 4 others (1985). A new tumor marker CA 15:3 defined by monoclonal antibodies. Initial clinical evaluation in patients with breast cancer. *Proc. XIII Annual Meeting ISOBM*, Paris (*Abstract*).

BOLLA, M., DAIDJ, H., LACHET, B., BOUCHET, Y. & VROUSOS, C. (1983). L'antigène carcinoembryonnaire dans le tumeurs du sein: Evaluation initiale et surveillance. *Bull. Cancer*, **70**, 300.

HILKENS, J., HILGERS, J., BUIJS, F. & 4 others (1983). Monoclonal antibodies against human milkfat globule membranes useful in carcinoma research. *Prot. Biol. Fluids*, **31**, 1013.

KUFE, D., INGHIRAMI, G., ABE, M., HAYES, D., JUSTIWHEELER, H. & SCHOLM, J. (1984). Differential reactivity of a novel monoclonal antibody (DF3) with human malignant versus benign breast tumors. *Hybridoma*, **3**, 223.

MILLER, A.B., HOOGSTRATEN, B., STAQUET, M. & WINKLER, A. (1981). Reporting results of cancer treatment. *Cancer*, **47**, 207.

NAMER, M., HERY, M., ABBES, M. & 5 others (1978). Interest of carcinoembryonic antigen in the follow-up and treatment of breast cancer. In *Clinical Application of Carcinoembryonic Antigen*. KREBS, *et al.* (eds), p. 225. Excerpta Medica, Amsterdam.

NAMER, M., KREBS, B.P., RAMEAU, Ph., HERY, M. & PHILIP, C. (1985). Les marqueurs tumoraux dans le cancer du sein: Comment les utiliser en 1985? In *Les maladies du sein en 1985: Formation médicale continue en Sénologie*. 7 ème journée de la Société Française de Sénologie et Pathologie Mammaire, Demaille (ed.) p. 20.

TOBIAS, R., ROTHWELL, C., WAGNER, J., GREEN, A. & V.LIU, Y.S. (1985). Development and evaluation of a radioimmunoassay for the detection of a monoclonal antibody defined breast tumor associated antigen 115D8/DF3. *Clin. Chem.*, **31**, 986.

TORMEY, D.C., WAALKES, T.P., AHMANN, D. & 4 others (1975). Biological markers in breast carcinoma. Incidence of abnormalities of CEA, hCG, three polyamines and three minor nucleosides. *Cancer*, **35**, 1095.

WANG, D.Y., BULBROOK, R.D., HAYWARD, J.L., HENDRICK, J.C. & FRANCHIMONT, P. (1975). Relationship between plasma carcinoembryonic antigen and prognosis in women with breast cancer. *Eur. J. Cancer*, **11**, 615.